# Obstetrics

**Published:** 1904-12-17

**Authors:** 


### OBSTETRICS.

**Eclampsia.**—Dr. R. N. Willson[1] discusses the significance of urinalysis in pregnancy, with special reference to eclampsia, and arrives at the following conclusions:—(1) Careful examination of the urine should be made in all cases of pregnancy, especially during the later months. (2) The presence of albumin, the diminution of the eliminated urea, and the presence of a microscopic renal sediment (casts, renal epithelium, blood, etc.) are important indications of impaired renal function. (3) Even if the urine appears perfectly normal the possibility of eclampsia must be considered, especially in young women. (4) When eclampsia supervenes upon labour in a patient with previously (apparently) healthy kidneys, the tendency subsequently is towards a return to normal renal function if the patient survives. (5) Until the nature and ultimate cause of uræmia and eclampsia are more thoroughly understood it would appear that urinalysis, though not an unerring guide, is our most valuable index of the condition of the kidneys and our most trustworthy source of information as to danger from such forms of toxæmia. (6) The prognosis seems to be vastly improved if eclampsia be combated by bleeding, followed by venous transfusion with normal salt solution. These measures reduce and dilute the poison in the circulation, and relieve the cardiac distress. Helme[2] records a case of eclampsia in which he performed spinal subarachnoid puncture, on the assumption that the convulsions and stupor are dependent on increased intracranial pressure. One drachm and a half of cerebro-spinal fluid was withdrawn, the fluid escaped rapidly, as if under considerable pressure. Marked improvement in the condition of the patient followed, and she ultimately completely recovered. Baldonsky[3] reports two cases of eclampsia successfully treated with thyroid extract; in one, however, narcotics were administered as well. Sir Halliday Croom,[4] after discussing the various theories of eclampsia, says that our knowledge of its causation and pathology is still very unsatisfactory. It is no doubt due to the presence of some toxic material in the blood, and the great point is to promote the elimination of the poison. He strongly advocates rapid delivery, as in the majority of cases the fits then cease. He records two cases in which he performed Cæsarian section, and says that this operation is justifiable when the mother is in extreme peril and it is impossible to deliver rapidly through the natural passages.

**Inversion of the Uterus.**—Dr. Robert Boxall[5] records a case of inversion of the uterus which occurred as a result of traction on the cord. An unsuccessful attempt at re-inversion was made within a few hours, and again 48 hours after delivery. Some adherent portions of placenta and membranes having been removed and the parts cleansed, it was decided to defer any further attempt for a few weeks. Hot douches were used night and morning, and when the patient was examined six weeks after the uterus was found to have regained its normal position. This probably occurred during the third week, as at that time there was a certain amount of abdominal pain, free red discharge, and pyrexia, which could not otherwise be accounted for. The case is one of considerable interest on account of the extreme rarity of spontaneous re-inversion.

**Puerperal Infection.**—J. W. Byers[6] makes the following practical suggestions for bringing the results of private midwifery work more into line with the brilliant records now achieved in properly conducted maternities. (1) Students and nurses should be taught clearly the risks of vaginal examination, and the great value and absolute safety of replacing this method by abdominal palpation; (2) It should be thoroughly impressed upon students and nurses that the organisms which cause puerperal infection are conveyed from without, and that unless the greatest care is exercised it is extremely easy for them to be introduced and the patient infected; (3) Whenever interference is necessary during the course of labour the same precautions should be taken as regards patient, instruments, and hands as are now employed in abdominal operations.

**Retroversion of the Gravid Uterus.**—Herman[7] maintains that in the majority of cases reposition will occur spontaneously if the bladder be kept emptied, and the patient kept in the recumbent position. When reposition is necessary he recommends that the uterus be pushed up with the fingers in the vagina; if this is unsuccessful the patient should be anæsthetised and pressure made on the fundus with two fingers in the rectum. He considers that the various instruments devised for the special purpose of pushing up the uterus are quite unnecessary and sometimes dangerous. For those cases in which the uterus is fixed in its abnormal position by adhesions, abdominal section has been advocated, but Herman considers that if the bladder be kept emptied, the reduction of the uterus is not necessary, as either the pregnancy will go to full term (the uterus enlarging at the expense of its anterior wall) or else spontaneous abortion will take place.

**The Treatment of Accidental Hæmorrhage.**—There is still considerable difference of opinion as to the best method of dealing with accidental hæmorrhage. In cases where it is associated with some constitutional disease, such as hæmophilia, scurvy, nephritis, and the like, Sir A. Macan [8] insists on the importance of prophylactic treatment. For the arrest of the hæmorrhage he favours plugging the vagina in preference to rupturing the membranes. This method of treatment is also recommended by Dr. Purefoy,[9] who says that the judicious use of the vaginal tampon controls bleeding, and initiates uterine action, the absence of which forms the chief source of danger to the patient's life. He thinks that the risk of internal hæmorrhage following plugging has been greatly exaggerated.

**Pyelitis Complicating Pregnancy.**—E. B. Cragin[10] reports ten cases of pyelitis complicating pregnancy. He agrees with Vinay that the etiology of the condition probably depends upon two factors:—(1) Compression of the ureter by the pregnant uterus; (2) infection of the urinary tract above the point of compression. The right kidney appears to be much more frequently affected than the left. The infecting organism is usually the colon bacillus, and the period of pregnancy at which the complication is most likely to occur is between the fifth and eighth month. The most prominent symptoms are (1) pain in the lumbar region, sometimes very acute, sometimes only elicited by palpation or motion; (2) a rise of temperature varying from 1·5° to 6·5°; (3) irritability of the bladder. On examination the affected kidney is usually felt to be enlarged and tender. The urine is acid; at first it may contain only a trace of albumin and perhaps a few casts, later pus cells, renal epithelium, and bacteria are present. One of the features of chief interest in pyelitis complicating pregnancy is the diagnosis; the conditions most likely to be confused with it are appendicitis, typhoid fever, and salpingitis. Of great importance in the diagnosis is the careful examination of the urine—chemical, microscopical, and bacteriological. The prognosis in most cases is favourable. The treatment consists of rest in bed, fluid diet (especially milk), and urinary antiseptics. For the relief of pain an ice-bag over the kidney, and if this fails to relieve an occasional opiate may be given. When in spite of this treatment there is evidence of extension of infection to the kidney substance, surgical interference by nephrotomy or nephrectomy is indicated. Interruption of the pregnancy is seldom if ever advisable. Leguen,[11] writing on the same subject, says that when the pyelo-nephritis occurs during the puerperium the diagnosis from puerperal infection may be very difficult. The principal distinguishing features are the great oscillations of temperature, the pulse not corresponding with the fever, and the general condition remaining better than in puerperal infection. With regard to treatment he says that in bi-lateral pyelo-nephritis, where the condition necessitates active intervention, the induction of premature labour or abortion is justifiable. In unilateral cases requiring active treatment he thinks nephrotomy should be the operation of choice, but that induction of labour may be necessary during the last month.

[1] Amer. Jour. of Med. Sci., Feb. 1904. [2] Brit. Med. Jour., May 14, 1904. [3] Brit. Gynæcol. Jour., Aug. 1904. [4] Lancet, June 18, 1904. [5] Ibid., July 16, 1904. [6] Ibid., Aug. 13, 1904. [7] Brit. Med. Jour., April 16, 1904. [8] Lancet, Aug. 6, 1904. [9] Rotunda Hospital Rep., June 1904. [10] Med Rec., July 16, 1904. [11] Jour. of Obstetric and Gynæcology of the British Empire, Aug. 1904.

